# Effects of the *Dobbs* decision on abortion and related service provision among sexual and reproductive health clinics in the United States: results from a qualitative study

**DOI:** 10.1080/26410397.2025.2557074

**Published:** 2025-09-08

**Authors:** Jennifer Mueller, Sidney Cech, Octavia Mulhern, Alicia VandeVusse

**Affiliations:** a Senior Research Associate, Guttmacher Institute, New York, NY, USA.; b Senior Research Assistant, Guttmacher Institute, New York, NY, USA; c Research Associate, Guttmacher Institute, New York, NY, USA; d Senior Research Scientist, Guttmacher Institute, New York, NY, USA

**Keywords:** abortion provision, policy change, US, Dobbs, sexual and reproductive health

## Abstract

Since the release of the *Dobbs v. Jackson Women's Health Organization* Supreme Court decision in June 2022, researchers have been working to better understand the impact that the growing number of abortion restrictions is having on the provision of and access to abortion services in the United States (US). Less is known about the impact of abortion restrictions on the provision of sexual and reproductive health (SRH) services more broadly, including at clinics that do not directly offer abortion. Between November 2023 and February 2024, we conducted interviews with SRH managers, clinic managers, and other administrators at publicly funded SRH clinics around the US to generate evidence on the effects of state-level abortion restrictions on the provision of abortion and related services, such as pregnancy options counselling and abortion referrals, at facilities providing contraceptive services. Through content analysis, we found that, while clinics in states with abortion restrictions have stopped providing abortions or are struggling to provide abortions amidst the variety of restrictive policies, clinics in states without these restrictions are also facing difficulties, specifically having to adjust their care provision due to the increase in patients seeking abortion. Simultaneously, the complicated web of abortion regulations has led providers in all states to experience challenges related to combating misinformation and protecting reproductive autonomy. These findings demonstrate the ways in which the ripple effects of *Dobbs* extend well beyond abortion and affect the wider network of publicly funded SRH care.

## Plain language summary

In 2022, the Supreme Court of the United States overturned the right to abortion. This has allowed states to pass their own policies restricting or protecting abortion. Clinics that receive funding from the government to provide sexual and reproductive health care are likely to be affected by these changing policies, since they provide abortions and referrals for abortion, counselling for patients who are pregnant, and treatment for people who are miscarrying a pregnancy. We interviewed staff at these clinics to better understand how their work has been affected by the changing policies. The staff in states that have fewer restrictions told us that they are changing the way they work to try to see as many patients as they can. The staff in states with more restrictions said that they are having a hard time providing abortion, counselling pregnant patients, and providing miscarriage treatment. All of the staff we talked to described how their clinics are working to continue to provide sexual and reproductive health care.

## Résumé

Depuis la publication par la Cour suprême de son arrêt sur Dobbs v. Jackson Women’s Health Organization en juin 2022, les chercheurs s’efforcent de mieux comprendre l’impact du nombre croissant de restrictions à l’avortement sur la fourniture des services d’avortement et l’accès à ceux-ci aux États-Unis d’Amérique. On connaît moins bien les conséquences des restrictions à l’avortement sur la prestation des services de santé sexuelle et reproductive (SSR) au sens large, notamment dans des centres qui ne proposent pas directement l’avortement. Entre novembre 2023 et février 2024, nous avons mené des entretiens avec des responsables de la SSR, des directeurs de centres de santé et d’autres administrateurs des centres de SSR financés par des fonds publics aux États-Unis afin de recueillir des données sur les effets des restrictions au niveau des États sur la fourniture de services d’avortement et des services apparentés, comme le conseil en matière de grossesse et l’orientation vers des services d’avortement, dans des établissements proposant des services de contraception. Grâce à une analyse de contenu, nous avons constaté que, si les centres des États appliquant des restrictions à l’avortement ont cessé de pratiquer des avortements ou peinent à le faire face à la diversité des politiques restrictives, les centres situés dans des États exemptés de ces restrictions rencontrent également des difficultés, notamment l’obligation d’adapter leur offre de soins en raison de l’augmentation du nombre de patientes souhaitant avorter. Parallèlement, la complexité des réglementations en matière d’avortement a conduit les prestataires de soins dans tous les États à éprouver des difficultés pour lutter contre la désinformation et protéger l’autonomie reproductive. Ces résultats démontrent que les répercussions de Dobbs vont bien au-delà de l’avortement et touchent le réseau plus large des soins de SSR financés par les fonds publics.

## Resumen

Desde la emisión de la decisión de la Corte Suprema en el caso *Dobbs c. Jackson Women's Health Organization* en junio de 2022, los investigadores han estado trabajando para entender mejor el impacto que la creciente cantidad de restricciones al aborto está teniendo en la prestación y accesibilidad de los servicios de aborto en Estados Unidos (EE. UU.). Menos se sabe sobre el impacto de las restricciones al aborto en la prestación de servicios de salud sexual y reproductiva (SSR) de manera más amplia: por ejemplo, en clínicas que no ofrecen aborto directamente. Entre noviembre de 2023 y febrero de 2024, realizamos entrevistas con administradores de servicios de SSR, administradores de clínicas y otros administradores en clínicas de SSR financiadas públicamente en EE. UU., con el fin de generar evidencia de los efectos de las restricciones al aborto a nivel estatal en la prestación de servicios de aborto y otros servicios afines, tales como consejería sobre las opciones de embarazo y referencias de aborto, en establecimientos de salud que proporcionan servicios de anticoncepción. Mediante el análisis de contenido, encontramos que, si bien las clínicas en estados con restricciones al aborto han parado de proporcionar abortos o tienen dificultades para proporcionar abortos a causa de la variedad de políticas restrictivas, las clínicas en estados sin estas restricciones también enfrentan dificultades, en específico por tener que ajustar su prestación de servicios debido al aumento de pacientes que buscan un aborto. A la vez, debido a la complicada red de normativas referentes al aborto, prestadores de servicios en todos los estados están enfrentando retos relacionados con combatir información errónea y proteger la autonomía reproductiva. Estos hallazgos demuestran las maneras en que los efectos dominó de *Dobbs* se extienden más allá del aborto y afectan más ampliamente la red de servicios de SSR financiados con fondos públicos.

## Introduction

In June 2022, the Supreme Court of the United States (US) overturned *Roe v. Wade* in the *Dobbs v. Jackson Women’s Health Organization* decision, eliminating the federal right to abortion and leaving abortion legality up to the states. Since then, abortion legality has been in flux as many abortion restrictions and protections have been passed, overturned, or blocked. As of April 2025, abortion is banned with very limited exceptions in 12 states, six states have bans at 6, 12, or 15 weeks of gestation, and 23 states have gestational duration bans at 18 weeks or later.^1^ Despite reductions in abortion access due to the *Dobbs* decision, there was an 11% increase in the number of clinician-provided abortions in the US in 2023 as compared to 2020.^2^ However, abortion access has been constrained and redistributed across the country because there are now far fewer places in the US where people can access these services, affecting the network of sexual and reproductive health (SRH) clinics that provide abortion and related care. Restricting access to abortion infringes on the fundamental human rights of people seeking essential health care.^3^

Abortion restrictions impact patients, as well as clinics and providers that offer abortion care, and interfere with people’s ability to achieve reproductive autonomy.^4^ People who are unable to access abortions face increased physical health risks associated with carrying a pregnancy to term, as well as higher rates of economic insecurity than those who receive abortions.^5,6^ One study found that people who travel for abortion care experienced negative emotions caused by having to explain their travel (to their friends, family, or employer), being away from their support network, and fear about legal repercussions.^7^ Nevertheless, out-of-state travel for abortions has increased since *Dobbs.*^8^ Consequently, SRH care providers in states with restrictions may be experiencing high rates of moral distress – when individuals feel “powerless to do what they think is right” – and declines in decision-making autonomy.^9–11^ Many providers may be concerned about their legal risk when making decisions about patient healthcare related to abortion and have faced uncertainty about whether they can continue to counsel patients on abortion.^12,13^ Previous research has highlighted how abortion restrictions lead to burnout, fatigue, and stress among those who provide abortion care.^13^ Furthermore, as a result of abortion restrictions, clinics may be adjusting the care they provide to patients in order to cope with an increase in out-of-state patients seeking abortion services.

Abortion restrictions also impact SRH clinics that do not directly offer abortion services, which is the majority of publicly supported SRH clinics (13% provide medication abortion and 4% provide procedural abortion).^14^ Across the US, millions of people rely on publicly funded SRH clinics for care.^15^ These facilities, which are supported by federal, state, or local funding, include Planned Parenthood clinics, federally qualified health centres (FQHCs), public health department clinics, hospitals, and independent clinics. Most of these clinics provide pregnancy testing, and many provide non-directive pregnancy options counselling and abortion referrals.^14^ Experts recommend that all SRH providers provide patients with accurate and unbiased information about pregnancy options and make any appropriate referrals.^16,17^ Moreover, discussion of all pregnancy options is associated with higher quality of care.^18,19^ A few studies have found that only a minority of patients report discussing all pregnancy options with their provider, though patients overwhelmingly expressed that providers should discuss parenting, adoption, and abortion.^18^ However, the increasingly hostile abortion environment in the US may constrain providers’ ability to adhere to this standard of care and ensure patients have access to necessary information to achieve reproductive autonomy. For example, clinics that receive funding from the federal Title X program (which is dedicated to supporting the delivery of family planning care) are required to offer pregnant patients the opportunity to receive factually accurate information and non-directive counselling regarding prenatal care and delivery; infant care, foster care, or adoption; and pregnancy termination.^20^ During the implementation of the “domestic gag rule” from 2019 to 2021, Title X sites were barred from referring patients for abortions and were required to physically and financially separate abortion services from Title X-funded services. These temporary restrictions led to many sites leaving the Title X program and were associated with reduced patient access to contraception.^21,22^ Little is known about how publicly funded family planning clinics and Title X clinics have adjusted their abortion care in response to restrictions associated with the *Dobbs* decision.

A third of publicly supported SRH clinics also provide some forms of miscarriage management,^14^ for which some of the treatment options are the same as those used for abortion. Patients experiencing early pregnancy loss have the option of expectant management (waiting for the loss to happen naturally, without treatment unless it becomes necessary) or active management (provision of medications or surgical intervention).^23,24^ However, miscarriage management has become more challenging in the wake of *Dobbs*, because “ambiguous and varied interpretation of an ever-changing legal landscape” may lead to confusion and fear among providers and patients.^24^ Due to the threat of legal consequences, many providers may feel compelled to modify their counselling and withhold interventions when treating patients experiencing miscarriage.^24,25^ While previous research has demonstrated that provision of this miscarriage care has clearly been affected by the increasing number and severity of abortion restrictions in the United States, few studies have explored the impacts of these restrictions at SRH clinics.

It is critical to investigate how clinics that provide essential SRH services and help people to exercise their reproductive rights have been affected by recent abortion restrictions. While past research has documented some of the negative consequences of abortion restrictions on patients and providers, less work has been done to understand how clinics that primarily provide contraception and other SRH services, especially those that are publicly funded, have been affected by abortion restrictions. This study builds on the existing literature to paint a clearer picture of the consequences of the *Dobbs* decision on providers and staff at publicly supported SRH clinics in both restricted and less restrictive/protective states.^10–13,24,26^ We aimed to explore how provision of abortion, options counselling, and miscarriage management, as well as referral for abortion, have been affected at these clinics since *Dobbs*.

## Methods

### Sample and recruitment

We recruited participants who completed the Guttmacher Institute’s 2022–2023 Survey of Clinics Providing Contraceptive Services and opted in to being contacted to participate in a follow-up interview.^14^ Participants included directors, managers, administrators and medical providers at publicly supported facilities that provide contraceptive services across the US*.* A purposive sampling design was used to narrow the list of potential participants, ensuring representation from facility staff with varied practice experiences in states with differing abortion policies. A few participants were also recruited through snowball sampling, where interviewees suggested colleagues from similar organisations for inclusion in the study.

We aimed to achieve balanced representation, with approximately half of participants from states where abortion is “restrictive”, including states classified as “restrictive”, “very restrictive” or “most restrictive” according to the Guttmacher Institute’s Interactive Map of US Abortion Policies and Access After *Roe.*^1^ Ohio and Wisconsin were also considered restrictive because of limited access to abortion during the fielding period.^27,28^ The other half consisted of participants from states where abortion is “less restrictive/protective”, including states classified as “protective”, “very protective” or “most protective” according to the map. A few participants worked with clinics in multiple states with differing abortion restrictions and, therefore, spoke to the effects of abortion bans on clinics in both restrictive and less restrictive/protective states. States were categorised based on their abortion policies as of October 2023 (the initiation of data collection); this categorisation does not reflect the current policy landscape due to ongoing changes to abortion policies. In addition, a variety of clinic types were included: FQHCs, health department clinics, hospital clinics and independent clinics, and Planned Parenthood clinics.

All potential participants were contacted by email and/or phone, introduced to the study, and invited to participate. If they agreed, a virtual interview was scheduled for a mutually convenient time. We reached out to staff at 59 clinics in total. We began conducting interviews while recruitment was ongoing; we achieved data saturation towards the end of recruitment and aimed the remaining recruitment at trying to ensure representation from all clinic types and state abortion groups.

### Data collection

Interviews were conducted between November 8, 2023 and February 14, 2024. Authors JM, OM, and SC conducted the interviews; they all identify as women, hold Master’s degrees, and are research staff at the Guttmacher Institute. Interviewers were trained in conducting qualitative interviews and practised conducting interviews with the guide used for this study. Because of our recruitment methods, participants were familiar with the researchers’ institution but had no direct prior contact with the interviewers prior to the interview. Interviewees were aware that the interviewers work at an organisation dedicated to advancing SRH and rights and supportive of abortion rights.

In October 2023, we conducted five pilot interviews with clinic directors, managers, and administrators from Planned Parenthood clinics and independent clinics from five states to test the interview guide. At the end of each interview, pilot participants provided feedback on the guide’s clarity, flow, length, and content, which informed revisions of the guide before formal data collection began. During the interview, participants were asked about their abortion service provision, how it may have changed since *Dobbs,* and what they may be doing differently since. In restrictive states, participants were asked how they handle patients seeking abortion services; in less restrictive/protective states, they were asked if they have any concerns about providing abortion services and about their protocols for out-of-state patients seeking abortion. In addition, participants were asked about their pregnancy options, counselling and abortion referral practices. Participants were also asked about the impact of the *Dobbs* decision on their staff (including burnout/morale, recruitment/retention, training/workflows) and patients (including demographic shifts, how patients are paying for care, confidentiality). Interviews were conducted via Zoom, averaging 51 minutes in duration; only the participant(s) and interviewer were present. Each participant was offered a $150 gift card as a token of appreciation for their time.

### Ethics

A verbal informed consent protocol was utilised; after describing the study to the potential participant, the interviewer asked if they wanted to proceed with participating. Only those who verbally agreed to participate proceeded with the interview. All data collection materials and protocols were reviewed by the Guttmacher Institute’s Institutional Review Board (DHHS identifier IRB00002197) and determined to be exempt from review as the study data were from health professionals who were being surveyed and interviewed as part of their professional responsibilities (exemption received on August 9, 2023).

### Analysis

The interviews were audio-recorded with voice recorders and through Zoom. Each audio file was transcribed by a third-party service, and transcripts were subsequently reviewed for accuracy and de-identified. Immediately following each interview, interviewers briefly summarised the content in a memo. We conducted a content analysis of participant responses, identifying and categorising the presence of certain themes and concepts. We deductively created a coding scheme *a priori* based on the literature and topics included in the interview guide. Applying this coding scheme, we used NVivo to code two transcripts as a team and then reviewed and addressed coding discrepancies. Each team member (all authors) then individually coded a set of transcripts, and the team met to discuss coding questions and issues, as well as revise the coding scheme where necessary. Following the first round of coding, additional sub-codes were generated, and the transcripts were coded a second time to capture a greater level of detail. Once the coding process was complete, reports for codes relevant to this analysis were generated by abortion restrictiveness (restrictive vs. less restrictive/protective). Matrices for each of the code reports were created, which allowed for the identification of key themes and sub-themes.

## Results

We conducted 36 interviews with participants from 35 clinics located in 25 states. Slightly more than half of the clinics were in restrictive states, while slightly less than half were in less restrictive/protective states, and two participants worked with clinics in multiple states (Table 1). Almost half of the participants were from Planned Parenthood clinics, one quarter worked at hospitals/other clinics, one fifth worked at health department clinics, and four participants were from FQHCs. More than half of clinics in our sample have never provided abortion (61%), while around one quarter provided abortion both pre- and post-*Dobbs*. Two clinics provided abortion only pre-*Dobbs,* and two clinics only post-*Dobbs*. Two-thirds of participants worked at clinics that received Title X funding.

**Table 1.** Sample characteristics

Characteristics		N	%
**Clinic type**	Planned Parenthood	16	44
	Health department	7	19
	Federally qualified health centre	4	11
	Hospital/other	9	25
	**Total**	**36**	**100**
**Abortion status**	Less restrictive/protective	15	42
	Restrictive	19	53
	Multiple	2	6
	**Total**	**36**	**100**

We identified three main themes on how abortion restrictions affected publicly supported SRH clinics around the US: clinics in less restrictive/protective states are making adjustments to their clinic procedures to accommodate increased abortion demand, clinics in restrictive states are facing challenges providing abortion and related services, and abortion restrictions have caused upheaval in how clinics function in all states.

### Clinics in less restrictive/protective states are making adjustments to clinic procedures to accommodate increased abortion demand

Several participants in less restrictive/protective states described efforts their clinics undertook to expand abortion services following the *Dobbs* decision. These efforts included expanding medical abortion services, adding telemedicine services for medical abortion, and increasing the number of abortion appointments they offered.

*“We now offer telemedicine medical abortion services, which we have not been able to offer prior […] we’ve just expanded our abortion care services as well, as far as [general anesthesia] that we offer. So that’s, I think, […] supported the influx of patients that we’ve been seeing.”* (Participant 214, less restrictive/protective state)

A few sites also mentioned expanding abortion-related services, such as their ability to serve patients experiencing miscarriage. This included improving access to the early pregnancy loss medications misoprostol and mifepristone in their clinic, training staff on treating miscarriages, and putting miscarriage management policies in place.

*“In the past, if we had a patient come in [with a] miscarriage, we tended to say you need to go to the ER or you need to see your primary [care provider], or whatever. It was more of a comfort level and not enough training, and now since [the medical director] is on board, we definitely feel, I should say our clinicians feel like they can handle that. So, we’re able now to handle [patients experiencing miscarriage].”* (Participant 206, less restrictive/protective state)

Participants in less restrictive/protective states attributed much of the increased demand for abortion to out-of-state patients seeking care due to the implementation of abortion bans in their states of residence.

*“And then we saw in [state] last year […] we were just flooded with patients coming from all over [seven restrictive states].”* (Participant 119, less restrictive/protective state)

Several participants described efforts their clinics undertook to accommodate this increased demand for abortion from out-of-state patients, such as trying to prioritise these patients or increasing the overall number of patients they can see.

*“Our abortion services [team] have had to change the way they provide services, like the volume they are trying to accommodate. […] we are surrounded by states with greater restrictions on abortions. […] So, just through the volume that we see, we are changing some things in abortion services, expanding, turning different rooms into ultrasound rooms, seeing if we can increase our volume in our centers.”* (Participant 201, less restrictive/protective state)

Participants noted that caring for out-of-state patients introduced unique challenges both for providers dealing with changing legal environments and for patients navigating the logistics of abortion seeking. Some participants referenced concerns about laws that restrict them from providing care to patients located in other states.

*“It’s also hard because patients are leaving and sometimes have questions or medical concerns once they go home, and because of state laws we’re unable to provide medical guidance to them once they are back home, and it’s also sometimes hard to find somebody that is willing to provide post-abortion care to individuals once they do go back home, and [to connect them with someone] I know that that’s an ally and somebody that the patient can feel comfortable disclosing what happened.”* (Participant 214, less restrictive/protective state)

In addition to making changes to their abortion care, a few sites in less restrictive/protective states pivoted away from other SRH care following abortion bans in nearby states to provide care to as many abortion-seeking patients as possible.

*“So, it used to be that there maybe would be five or six medication abortion appointments in a given day and the rest would be family planning and then, since Dobbs, we have asked all of those sites to offer an entire day of medication abortion.”* (Participant 107, less restrictive/protective state)

Clinics in less restrictive/protective states described longer wait times for a variety of services, including Pap smears, IUD insertions, gynaecological care, and other routine SRH services due to changes in abortion demand and the prioritisation of abortion care due to its time-sensitive nature.

*“[Wait times increased] a tad bit. Only because this health center sees both reproductive health services, which is our abortion services in conjunction with what we call family planning, which is our normal, ‘I want an implant, I want a Depo, I want to start birth control visits.’ So, with the increase in both services […] I can say that the wait times might have increased.”* (Participant 118, less restrictive/protective state)

Moreover, some sites that do not provide abortions reported increased wait times for other SRH services due to an overflow of patients from sites that had pivoted towards abortion provision in the wake of *Dobbs*.

*“We are seeing overflow from, I think, patients who might otherwise go seek care at [other clinics] for non-abortion related services but now don’t have the capacity to pay or don’t want to wait for an STI treatment, STI testing, birth control refills. […] So, I think when folks are coming from out of state because they have no other option, that’s pushing some of our local patients for longer wait times and [it’s] harder to get those services as well.”* (Participant 226, less restrictive/protective state)

### Clinics in restrictive states are facing challenges providing abortion and related services

Several participants in restrictive states noted that they stopped providing abortion as a direct result of state abortion bans implemented following *Dobbs*.

*“We have closed all of our abortion health centers. Now, we had a couple of [family planning] centers that are co-located with abortion centers […] So, those centers that are co-located, those [abortion] facilities are just not operating. So, they are empty. Just very sad. Very sad, but the family planning centers are operating.”* (Participant 113, restrictive state)

For sites in restrictive states still providing abortion services at the time of the interview, participants mentioned a proliferation of abortion restrictions that complicate the provision of abortion care, such as mandatory waiting periods for patient consent, in-person requirements for consent appointments (rather than allowing them to happen via telehealth, for instance), and compulsory ultrasounds for dating the pregnancy.

*“[State] has the state law of the 24-hour waiting period law, so we have to see our patients twice […] The other state law that determines my scheduling is that the patients have to see the same provider if they do a medical abortion for day one and day two. So, it’s a little bit of a puzzle that we put together to schedule.”* (Participant 130, restrictive state)

Furthermore, participants in restrictive states described difficulties they encountered when providing or referring for miscarriage management post-*Dobbs*, such as fear that patients experiencing miscarriage will be referred to a provider who may not provide the necessary treatment.

*“So, something that I’ve thought about is if this patient starts to have excessive bleeding and I need to refer her to a provider to take care of her, who am I going to refer her to and how is this situation going to be handled, because I don’t think I need to … you know what I’m trying to say. It’s going to be … is the provider that she sees that I refer her to going to be able to help her? Is that provider going to be able to terminate the pregnancy, if that’s necessary, or are they going to have to send her to another state, or … ?”* (Participant 128, restrictive state)

In addition to abortion care, some participants in restrictive states highlighted ways in which they have had to change how and whether they provide options counselling in the wake of *Dobbs* due to restrictions on what information staff can relay to patients about abortion.

*“We are allowed to offer a list of providers who offer prenatal, like OBGYNs or something, and if those people offer abortions as well, they are allowed to be on our list, but we are not allowed in [state] to tell people that abortions are provided in those offices, and there is no abortion provider in [state].”* (Participant 232, restrictive state)

Several participants also noted that regulations pre-dating *Dobbs* already restricted their ability to assist pregnant patients seeking abortion, such as state laws prohibiting abortion referrals or the Title X gag rule, while others described how the lack of nearby abortion clinics stymied their ability to counsel pregnant patients effectively.

*“This was before Dobbs. Because of [state], we had to change wording in some of our referrals because we could not discuss abortion. We are not allowed to give that as an option to our patients even if they were to ask us. […] So, even if they were to say, “What about an abortion?” we are taught to say, “I am sorry, we are unable to talk about that, but here is a referral for our nurse-midwives or adoption services.” So, we don’t discuss abortion at all.”* (Participant 331, restrictive state)

A couple of participants further noted that their inability to provide comprehensive options counselling for pregnant patients, due to post-*Dobbs* abortion restrictions, contradicted their medical training.

*“We can’t even say the A-word or if a patient were to call in and say, “I am pregnant. Do you offer abortion services?” or “Can you help me with abortion services?” That’s just something we can’t discuss or give referrals for. We absolutely can’t offer them that and as a medical provider, [it’s like] being stuck between a rock and a hard place. We took an oath to provide the community education and awareness, and options. Now, because of the laws changing, we cannot offer that to them.”* (Participant 327, restrictive state)

### Abortion restrictions have caused upheaval in how SRH clinics function in all states

Although there were distinct themes that arose for restrictive versus less restrictive/protective states, many challenges of providing care after *Dobbs* cut across states. In particular, the need to urgently assist patients seeking abortion, concerns about patient confidentiality, rising misinformation and disinformation about abortion, and staff fear of prosecution and continued threats to SRH care were expressed by participants across states. Many participants from clinics in less restrictive/protective and restrictive states reported that their facilities increased abortion navigation services and related supports for patients because of the clear burden that restrictions on abortion posed for patients. These include connecting patients to financial support such as abortion funds, providing logistical assistance with travel, and offering discounted services.

*“We have launched a pretty robust patient practical support program for both in-state and out-of-state patients that’s predominantly funded by a grant and individual donors to support that cost.”* (Participant 107, less restrictive/protective state)

While clinics in less restrictive/protective states described efforts to support patients coming to their state for abortion care, those in restrictive states had abortion patient navigators who work to connect patients to out-of-state care. Clinics that were part of larger affiliates, in particular, described how they were able to leverage connections between affiliates to facilitate connecting patients to needed care.

*“So, we do have abortion patient navigators who assist with a lot of patients either going to another state for these abortion services or coming into state in helping with cost of the procedure or hotel or gas, or things like that. So, we do have the patient navigators in those roles to help, and they’ve established relationships with other affiliates as well.”* (Participant 130, restrictive state)

The need for increased abortion navigation was in part due to the pressure of increasingly stringent limitations on gestational duration imposed in many states in the wake of the *Dobbs* decision.

*“When a patient is ambivalent, some of it depends on how far along we think they are. You never want a patient to feel rushed but unfortunately, there are timelines where you might have a window that’s closing if you’re thinking about a medication abortion or even a procedural abortion. So, I try to be clear with them. A lot of patients don’t know that. How would they know if they haven’t been in that situation? So, I try to be clear with them as far as the window in which they have to make a decision […] So, being very clear about, ‘I want you to take the time that you need but if you think you might want this, you should decide by this time.’”* (Participant 226, less restrictive/protective states)

When counselling pregnant patients who expressed interest in abortion, participants frequently described having to contend with limits on gestational duration instituted both at the state level as well as by clinics offering abortions, which may implement more stringent gestational limits than required by law. These gestational limits introduce a sense of acute urgency to the decision-making process for pregnant patients and increase providers’ feelings of stress regarding these conversations.

*“I think we're just trying to communicate, as soon as patients call, you know, that time is of the essence.”* (Participant 119, multiple states)

In addition to abortion-related issues, several participants across both less restrictive/protective and restrictive states noted that their clinics instituted or upgraded their procedures for protecting patient privacy as a result of the *Dobbs* decision, and took steps to ensure that patients were educated on confidentiality. These concerns were particularly acute when providing abortion care to patients who travelled from restrictive states, as providers expressed worry that an electronic health record documenting abortion care could endanger a patient in the future.

*“I do have concerns for patient safety and patient confidentiality. For instance, we have patients that come from banned states that get care. Because we all use electronic medical records, the electronic medical records talk to each other and it can be very hard to shield people if they are concerned about disclosing that information when they go back to their state, the outcome of their pregnancy since that information sometimes is communicated inadvertently through the medical health record.”* (Participant 214, less restrictive/protective state)

Clinic staff also brought up having to contend with patient misinformation about abortion. Several participants from less restrictive/protective and restrictive states discussed the increasing presence and influence of crisis pregnancy centres – facilities that claim to be reproductive health care clinics but aim to dissuade people from accessing abortion^29^ – as sources of misinformation for their patients.

*“We have a ton of crisis pregnancy centers in Wisconsin close to some of our clinics that spew information that is just completely inaccurate. They have social media campaigns that are inaccurate about contraceptive methods or sexual health.”* (Participant 204, restrictive state)

Participants also described social media as a major source of misinformation about contraception and abortion for their patients.

*“I definitely see that in my patients who more and more only get their information from social media and will show us some of the stuff that they are seeing that is very compelling and convincing, but just frankly inaccurate.”* (Participant 226, less restrictive/protective state)

In response to the rise of mis- and disinformation, and to ensure that patients receive accurate information, one participant representing multiple clinics within a larger affiliate described their network’s efforts to actively combat misinformation.

*“[…] what we’re trying to do is more managed misinformation, bad information as an entire coalition in the states where it’s legal and also where it’s not. Just doing our best to make sure that the information that’s available to people is good information. Whereas, while we may not offer that specifically, I think as part of our larger advocacy efforts, marketing efforts, education efforts, we are trying to combat misinformation and bad information.”* (Participant 216, multiple states)

Restrictions on abortion care also led providers to experience fear and concern of legal consequences when counselling patients interested in abortion. This includes fear about what they can and cannot say or what information they are able to give patients.

*“I guess we don't automatically feel like we're protected. So, we always try to be proactive about making sure that we have the right attestations in place and making sure that our providers are not doing anything that could put them at risk. […] But with this, we have definitely, I think, been much more cautious about how we're approaching everything so that our physicians in particular are not persecuted in any way.”* (Participant 119, multiple states)

*“We would have so much to lose professionally and in multiple ways if we were sued because of being implicated to any extent in a termination. That’s just a lot to wrap your head around when you see patients who are making these really personal, sometimes really difficult decisions, and knowing that it could have this crazy repercussion on the team. […] And then, to have an extra layer of concern about how could this actually hurt us and end up with legal implications towards us, it’s hard.”* (Participant 102, restrictive state)

This fear and uncertainty contribute to staff burnout and low morale. Participants described the emotional toll of caring for patients in tough circumstances who may not be able to get the care they need.

*“For our abortion staff, the residual secondary trauma continues to be an area that we are trying to explore and do better on in terms of how we provide some resiliency and decompression opportunities for any staff working in abortion care, in transgender care, in family planning. It is all of it. I think it is most acute in our procedural abortion or medication abortion, particularly when patients are traveling long distances and having really complicated situations and needing to care for them.”* (Participant 107, less restrictive/protective state)

## Discussion

Our findings indicate that SRH clinics are trying to continue to provide high-quality care to meet patient needs and ensure reproductive autonomy even as regulations complicate their ability to do so. We found that clinics in less restrictive/protective states are responding to an increased demand for abortion by expanding access to medical abortion and telehealth services. This finding is consistent with research that has found an increase in overall abortion provision over the last several years.^2,30^ It is likely that the increased availability of telehealth medical abortion has contributed to the overall increase in abortions in the US, as telehealth may have improved access for people in rural areas or those who may not have been able to travel to seek services.^2,30,31^ Providing care via telehealth and offering medical abortion are ways to make care more patient-centred and meet patient preferences,^32,33^ with users of telehealth citing its privacy and expediency as benefits.^34^ However, there have recently been attempts to specifically restrict medical abortion, especially mifepristone, threatening access to an essential medication. Restrictions on telehealth for medical abortion have also been implemented; eleven states require at least one trip to the clinic to access medical abortion as of November 2024.^31^ In contrast, shield laws have been adopted in several states, which can protect providers who prescribe and mail medical abortion pills across state lines, while federal protections for mifepristone’s approval and availability ensure that it can continue to be prescribed and mailed.^35,36^ In this bifurcated policy context, we have found that providers in less restrictive/protective states are stepping up their efforts to increase abortion access for all patients, regardless of the abortion policy in their state of residence. Continuing education for providers located in shield law states about the extent of legal protections afforded to them by the law may help to expand access further.^37^

As demand for abortion increases in less restrictive/protective states, clinics in these states are dealing with the unique challenges of caring for out-of-state patients. Out-of-state travel for abortion has doubled in the last four years, with one-fifth of abortion seekers travelling to receive care.^8^ While travel to protective states is a way for people in restrictive states to access the care they need, travel adds logistical, emotional, and financial obstacles for patients and providers. Furthermore, anti-abortion advocates in some states have gone so far as to attempt to restrict travel for abortion, an unprecedented attack on people’s ability to access needed healthcare.^38^ In response, we found that clinics are working to provide care under abortion bans by increasing abortion navigation services and financial support that provide critical resources for abortion patients, especially for those who must travel out-of-state. The National Network of Abortion Funds reports a 39% increase in requests for support in the year following *Dobbs,* and that abortion funds financially supported over 100,000 individuals seeking abortions.^39^ While clinics and other practical support organisations often rely on donations in order to provide such support services, these funds may not be sustainable in the long term, and already the need for increasing support is making abortion even harder to access.^2,39^ Clinics seeking to support abortion access should continue to invest in abortion navigation services to assist patients travelling for care.

In addition, we found clinics in less restrictive/protective states struggling to balance the provision of abortion and other SRH services in the face of this increased demand. Our participants discussed how wait times for other types of SRH care have increased due to the prioritisation of abortion appointments. Minimising wait times for abortion care is critical given the proliferation of gestational duration abortion bans and the fact that over one-third of people accessing abortion discovered they were pregnant at six weeks or later.^40^ People seeking abortion have been reporting a wide variety of structural barriers in seeking these services, including gestational age limits, limited appointment availability, and long travel.^41^ However, it is also important that the prioritisation of abortion services does not inhibit the provision of other SRH care. Our previous research has found that these clinics are employing strategies such as expanding same-day appointments, expanding telehealth appointments, shifting to more flexible scheduling, and other changes to workflows for contraceptive provision in order to reduce wait times for other SRH services amidst the increased demand for abortion services.^26^ Overall, these clinics and providers are finding ways to serve more people in this time of need. Crucially, financial support for SRH services, such as Title X funding and state family planning programmes, is essential to maintaining access to care. Title X has been chronically underfunded, with stagnant funding since 2015, and is currently under threat from the Trump administration.^42^ To ensure high-quality SRH care is provided equitably and individuals are able to exercise their right to reproductive healthcare, funding for SRH must be restored and expanded.

Meanwhile, clinics in restrictive states reported challenges in providing abortion services. These clinics have already weathered a number of restrictions before *Dobbs* on abortion provision and referrals and options counselling, including the Hyde Amendment (which bans the use of federal funds for abortions),^43^ the Title X domestic gag rule,^44,45^ and targeted regulation of abortion providers (pre-*Dobbs* limitations on abortion that aimed to limit access).^46^ A growing body of evidence demonstrates that restrictions targeting abortion inevitably have spill-over effects on other forms of SRH care.^47^ Our study builds on this evidence to demonstrate how clinics in restrictive states are constrained in providing pregnancy options, counselling and referrals for abortion due to abortion bans. Some providers explained that their inability to provide counselling on all options conflicts with the medical training they have received, as limiting abortion referrals and pregnancy options counselling threatens person-centred care and reproductive autonomy by coercing people into unwanted medical decisions and care.^16,48^ Limiting the options presented to pregnant people leads to real harms, such as interfering in the patient-provider relationship and delaying people’s access to needed care. Advocates and clinicians must continue to advocate for providers’ obligation to fully inform patients of their options and for patients’ rights to fully informed consent.^16^

While only a small percentage of clinics in our sample provide abortion, the effects of abortion restrictions extend beyond access to abortion itself, limiting the provision of related reproductive health services, such as options counselling, miscarriage management, and referrals. Participants from clinics in restrictive states described difficulties providing or referring for miscarriage management since *Dobbs*, mostly out of concern for criminalisation if it was perceived that they had supported an abortion. Past research has found that medical institutions in hostile states are less likely to intervene with the full range of treatment options for miscarriages. Bans may lead to an increase in expectant management given concerns about criminalisation of abortion provision,^25,49^ with devastating consequences in some cases, including the deaths of women denied care in Texas emergency rooms.^50^ Other types of providers, like maternal-fetal medicine physicians or genetic counsellors, also describe facing restrictions and confusion about the pregnancy-related care they can provide post-*Dobbs.*^51,52^ We found that clinics and staff in these states are trying to continue to provide quality care despite being severely hamstrung by restrictions that have ripple effects on various areas of care. Protections for miscarriage management medications such as mifepristone and misoprostol would help ensure that clinics can provide this essential care without concerns about criminalisation.

In addition, participants across protective and restrictive states are changing clinic procedures in response to abortion restrictions. This includes dealing with misinformation and disinformation about abortion legality and safety. Abortion misinformation can divert people from care by perpetuating stigma and stoking fears about complications or side effects.^53,54^ Participants described misinformation coming from sources like social media or crisis pregnancy centres (CPCs). Many CPCs give inaccurate information to patients regarding the risks of abortion, such as links between abortion and breast cancer, mental health issues, and infertility.^55,56^ On social media, posts promote the use of ineffective or dangerous abortion procedures, along with perpetuating myths about risks and side effects.^57,58^ This increasingly prominent misinformation has further burdened clinics that now have to devote scarce resources to strengthening messaging and clinic materials in addition to devoting clinic time to addressing these beliefs. The more patients are misinformed, the more the burden of correcting misinformation and debunking myths is shifted onto providers, who are already stretched thin (as evidenced by the burnout and low morale that our participants noted). More research should be done on the effects of SRH misinformation on the receipt of, access to, and quality of care. Furthermore, legislation that prohibits deceptive advertising requires centres to disclose if they are licensed or makes public funding contingent on providing medically accurate information could help regulate CPCs and ensure patients have access to high-quality SRH care.^59^

Our participants also mentioned dealing with feelings of moral distress and concerns about legal consequences for providers offering abortion-related services. Prior research has found that providers in restrictive states report higher levels of moral distress about providing SRH care.^10^ Additionally, fears about providing abortion and related services in a restrictive climate can impact provider attrition and worsen SRH care deserts,^13,60^ deepening pre-existing deficits within the publicly supported SRH clinic network and exacerbating racial disparities in maternal and infant mortality.^44,61,62^ Previous research has also shown that abortion restrictions force SRH providers to choose between providing person-centred care and avoiding legal risk.^11,24,63^ This is particularly problematic given the growing recognition that person-centredness should guide SRH care.^17^ As abortion restrictions increase, greater numbers of people may be seeking to self-manage their abortions. In response, clinics may need to be prepared to educate staff on self-managed abortion pathways, resources to provide for patients, and potential complications, in addition to the legal risks of and protections for supporting patients in restrictive states.^64^ Thus, our study adds to the growing literature demonstrating that abortion restrictions impede efforts to ameliorate historical inequities by preventing providers from enacting patient-centred practices due to legal restrictions, severely limiting sexual and reproductive rights. Despite these ongoing challenges, providers and clinics continue to prepare to weather future attacks on reproductive health care. Federal protections for abortion, as well as other policy changes to address bans on essential health care, are needed to combat these ripple effects.

### Limitations

Our study is not without limitations. Our sample included participants who held a variety of roles at their clinics, both administrative and clinical; therefore, reports on clinic processes or care provision may be limited depending on the participant’s role. In some cases, participants were reporting on behalf of several clinics they work with, while others were only reporting on one clinic; there is likely variation that we missed across clinics and providers. The sample was also limited to those who opted into an interview, and therefore, our results may not represent clinics that are too overwhelmed by upheavals to participate or, conversely, do not have many changes to report. Participants may have answered questions in a socially desirable way to reflect that they are providing high-quality care despite the challenges, especially given that the interviewers were from the Guttmacher Institute (which is known to be supportive of abortion rights). Furthermore, given concerns around data security and privacy that participants named, some clinics may have chosen not to participate or to censor their responses. While we included clinics from many different states and clinic types, this sample is not representative of the publicly supported SRH clinic network in the US.

## Conclusion

Our study of SRH clinics across the US found that clinics in both less restrictive/protective and restrictive states are feeling the impacts of the *Dobbs* decision. Clinics in less restrictive/protective states are trying to meet the increased demand for abortion services, while the scope of services that clinics in restricted states can provide has narrowed. We found that the *Dobbs* decision, by impacting access to abortion and related services, is restricting people’s sexual and reproductive rights and exacerbating longstanding inequities in SRH care. SRH clinics continue to be essential sources of care for all people seeking SRH care, including those seeking abortion services. More research is needed to determine what the longer-term impacts of *Dobbs* will be on these service providers and how clinics will continue to adapt.

## Author contributions

*Conceptualsation*: *JM, AV*. *Data curation*: *JM, SC, OM, AV*. *Formal analysis*: *JM, SC, OM, AV*. *Writing – original draft*: *JM, SC, OM, AV*.

## Data Availability

In order to safeguard the confidentiality of our study participants, we are unable to make the data used for this study available for sharing.

## Provenance statement

This paper was not commissioned and went through external peer review.
